# Combined Activation of Guanylate Cyclase and Cyclic AMP in Lung Fibroblasts as a Novel Therapeutic Concept for Lung Fibrosis

**DOI:** 10.1155/2019/1345402

**Published:** 2019-03-07

**Authors:** Christopher Lambers, Panja M. Boehm, Yasemin Karabacak, Eslam Samaha, Alberto Benazzo, Peter Jaksch, Michael Roth

**Affiliations:** ^1^Division of Thoracic Surgery, Department of Surgery, Medical University of Vienna, Austria; ^2^Center for Molecular Biology, University of Vienna, Austria; ^3^Division of Respiratory Medicine, Department of Internal Medicine II, Medical University of Vienna, Austria; ^4^Pulmonary Cell Research, Department of Biomedicine and Pneumology, Department of Internal Medicine, University Hospital and University of Basel, Switzerland

## Abstract

Remodelling of the peripheral lung tissue and fibrotic foci are the main pathologies of idiopathic pulmonary fibrosis (IPF), a disease that is difficult to treat. TGF-*β* activation of peripheral lung fibroblasts is indicated as the major cause of tissue remodelling in IPF and is resulting in fibroblast hyperplasia and deposition of extracellular matrix. Soluble guanylate cyclase (sGC) stimulators combined with cyclic AMP (cAMP) activators have been reported to reduce proliferation and matrix deposition in other conditions than IPF. Therefore, this drug combination may present a novel therapeutic concept for IPF. This study investigated the effect of BAY 41-2272 and forskolin on remodelling parameters in primary human lung fibroblasts. The study determined TGF-*β* induced proliferation by direct cell counts after 3 days; and deposition of collagen type-I, type III, and fibronectin. BAY 41-2272 significantly reduced TGF-*β* induced fibroblast proliferation, but did not reduce viability. This inhibitory effect was further supported by forskolin. Both BAY 41-2272 and forskolin alone reduced TGF-*β* induced collagen and fibronectin* de novo* synthesis as well as deposition. This effect was significantly stronger when the two compounds were combined. Furthermore, the TGF-*β* induced expression of fibrilar *α*-smooth muscle actin was reduced by BAY 41-2272 and this effect was strengthened by forskolin. In addition, BAY 41-2272 and forskolin reduced TGF-*β* induced *β*-catenin. All effects of BAY 41-2272 were concentration dependent. The findings suggest that BAY 41-2272 in combination with cAMP stimulation may present a novel therapeutic strategy to reduce tissue remodelling in IPF.

## 1. Introduction

Idiopathic pulmonary fibrosis (IPF) was considered a rare disease that was diagnosed mainly in patients older than 50 years of age [1, 2]. However, a recent analysis indicated that its prevalence is increasing and equals today that of stomach, liver, testicular, and cervical cancers [3]. Worldwide, about 5 million people suffer from IPF with 12 per 100,000 newly diagnosed cases per year [4]. The cause of IPF is unknown, but smoking and viral infections, as well as the family history of IPF may be involved.

Diffuse tissue remodelling of the alveolar wall is the major pathology of IPF and seems to be resistant to available therapies [1, 5]. Studies in human and animals implied that transforming growth factor- (TGF-) *β*1 is a major cause of tissue remodelling in IPF and acts through tyrosine kinase receptors [6]. Currently, two drugs are approved for IPF therapy and both act by blocking tyrosine kinase receptors; however, their effect on tissue remodelling is drug specific [7, 8].

Isolated human IPF derived fibroblasts expressed markers, characterising the cells as myofibroblasts [9]. IPF fibroblasts are less flexible, express fibrilar collagen, and respond stronger to TGF-*β*1 stimulation compared to nondiseased fibroblasts. Furthermore, IPF derived fibroblasts produced a disease specific extracellular matrix (ECM), which modified the behaviour of nondiseased fibroblasts. As a result, healthy fibroblasts seeded onto ECM produced by IPF fibroblasts, expressed *α*-smooth muscle actin and phosphorylated Smad3 and STAT3. Furthermore, IPF derived ECM increased proliferation and migration of nondiseased fibroblasts [7]. These data suggest that the ECM produced by IPF fibroblasts must contain active TGF-*β* and may explain the earlier described increase of TGF-*β* and its receptors in IPF [10].

The ECM produced by IPF derived fibroblasts showed a disease specific modified expression of collagen type I, type III, and type V and fibronectin. The expression of all three ECM components was reduced by pirfenidone and nintedanib, however, in a drug-specific pattern [8]. In IPF, the stimulatory effect of the local composition of the ECM on fibroblast proliferation can either result from the mitogenic effect of collagen type I as shown in nondiseased human lung fibroblasts [11] or from the direct proproliferative effect of TGF-*β*1 released from alveolar epithelial cells [12]. Furthermore, IPF derived fibroblasts were less sensitive to collagen type I induced cell death, which was explained by the lack of Akt/mTOR signalling [13]. Together, these studies suggested that IPF may result from faulty signalling of resident fibroblasts responding to the modified ECM surrounding them [14]. The two drugs available for IPF therapy, pirfenidone and nintedanib, improve lung function [15], but have different effects on remodelling parameters of IPF patients, indicating that additional therapeutic options should be investigated.

In animal models of other types of fibrosis, soluble guanylate cyclase (sGC) stimulators reduced fibrotic events. The* de novo* synthesis of collagen type I was reduced by sGC due to the inhibition of TGF-*β*1 induced Erk1/2 signalling in human lung fibroblasts [16, 17]. In pulmonary hypertension, the sGC stimulator, BAY 41-8543, reduced vessel wall fibrosis [6, 18]. BAY 41-2272 reduced vascular remodelling and right heart ventricular hypertrophy, which resulted from profibrotic remodelling [19]. These studies suggested that sGC are a new class of drugs that could be used in IPF therapy.

The role of cyclic-AMP (cAMP) in the pathogenesis of IPF had been investigated in a few studies and showed its interaction with TGF-*β* signalling [20, 21]. In IPF derived fibroblasts, cAMP activation reduced proliferation and ECM deposition [22]. In bronchial epithelial cells of COPD patients, the activation of cAMP by other classes of drugs also prevented TGF-*β* induced remodelling and epithelial-mesenchymal transition [23].

In this study, the effect of BAY 41-2272 alone and in combination with cAMP activator (forskolin) on fibroblast proliferation and deposition of ECM components was investigated.

## 2. Methods

Nondiseased human lung fibroblasts (CC-2512) were obtained from Lonza, Switzerland, and were grown using standard protocols. Experiments were performed in 70%-80% confluent cell cultures between passages five to eight. Fibroblasts were grown in PRMI-1640 supplemented with 10% fetal calf serum, 20 mM HEPES, 8 mM L-glutamine (GlutaMAX), and 1 x nonessential amino acid mixture (all: Gibco/BRL, Thermo Fisher Scientific, Switzerland). Cells were characterised by their long stretched spindle phenotype which stained positive for fibronectin and inducible staining for *α*-smooth muscle actin (*α*-SMA).

### 2.1. Cell Proliferation and Cell Viability

Cell proliferation and cell viability were determined by direct cell count using Nexcelom Cellometer T4 © (Nexcelom Bioscience, Lawrence, Massachussetts, USA) and Trypan blue staining after 24 and 72 hours of incubation according to the manufacturer's protocol [24].

### 2.2. Quantitative Real-Time PCR

Total mRNA was isolated using the TRIZOL standard protocol and directly transcribed into cDNA using a reverse transcriptase kit (Advantage RT-for-PCR Kit, Clontech, BD Biosience, Palo Alto, USA) according to the manufacturer's instructions. Real-Time PCR was performed using a LightCycler480 (Roche Diagnostics, Mannheim, Germany) to determine mRNA of the following genes: collagen type I and type III, fibronectin, *α*-SMA, and *β*-catenin (TaqMan gene expression assays), Applied Biosystems (ABI, CA, USA). PCR conditions were 45 cycles with 95°C for 10 sec, 65°C for 30 sec, and 72°C for 5 sec. Human18s or Actin TaqMan Predeveloped Assay (ABI, UK) was used as control. The target gene was normalized against the reference gene (18s/ACTIN mRNA) and data was expressed as relative increase or decrease from baseline values. Relative mRNA expression was determined using the ΔCT method. ΔCT indicates the difference between the amplification cycles of the target and the housekeeping gene [24].

### 2.3. Protein Expression and Extracellular Matrix Deposition

Deposition of collagen types I and III and of fibronectin was determined using a cell based ELISA as described earlier [24]. All antibodies were obtained from Santa Cruz Bio Technology (Santa Cruz, USA; COL1A1 SC-8784, COL3A1 sc-271249, fibronectin SC-6952) and diluted 1:5'000 in blocking buffer. Secondary antibodies (Santa Cruz Bio Technology) were species specific and diluted 1:1'000 in blocking buffer; incubation was 1 hour (room temperature). After 3x washes with PBS optical densitometry was performed by ELISA reader (Thermofisher Scientific).

Structure and protein levels of *α*-SMA and *β*-catenin were determined by immunocytochemistry in cells which were grown on cover slips after 24-hour treatment. Cells were washed once with PBS, fixed in 4% formalin (2x5 minutes), followed by 1x wash with PBS, blocking in PBS, 0.01% Tween 20, 2% bovine serum albumin (30 minutes), before being incubated overnight (4°C) with the first antibody for *α*-SMA (Santa Cruz Biotech, sc-53015) or for *β*-catenin (Santa Cruz Biotec, sc-59737). After 3x washes with PBS, slides were incubated with a FITC labelled antibody (30 minutes, room temperature), nuclei were stained by DAPI, and pictures were obtained after 3x washes in PBS by EVOS FL cell imaging system (Thermofisher Scientific, Switzerland).

### 2.4. Statistical Analysis

Data are presented as mean ± standard deviation (SD). The null hypothesis showed no difference between treated and untreated cells. Protein expression and mRNA transcription were compared by Student's t-test (two-tailed, paired). Results were considered statistically significant with p value < 0.05.

## 3. Results

### 3.1. Combined sGC and cAMP Activation Prevented TGF-*β*1 Induced Cell Proliferation

**Figure 1:**
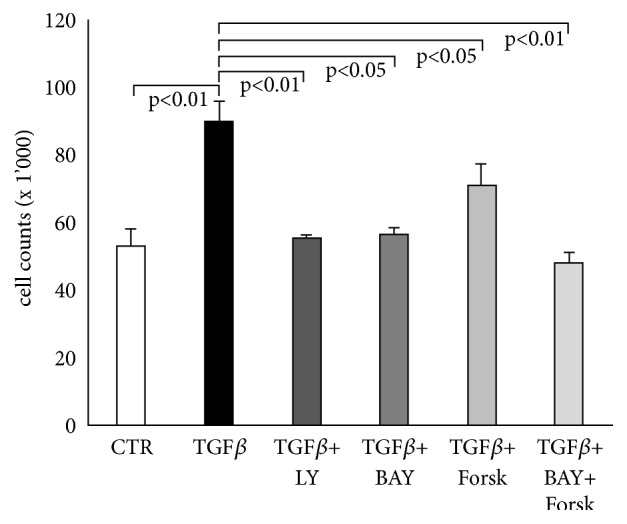
sGC and cAMP activation reduce TGF-*β*1 stimulated fibroblast proliferation. Fibroblasts were preincubated for 30 minutes with either BAY 41-2272 (10^−6 ^M), LY2157299 (10^−6 ^M) or forskolin (10^−6 ^M) before being stimulated with TGF-*β*1 (10ng/ml) for 3 days. Cell counts were performed in triplicate and bars represent the mean ±SEM. P values were calculated by paired Student's t-test.

Stimulation of human lung fibroblasts with TGF-*β*1 significantly increased fibroblast proliferation up to 1.7-fold (p<0.01) within 72 hours, as compared to unstimulated cells (Figure 1). The mitogenic effect of TGF-*β*1 was completely reduced by the TGF-*β*1 inhibitor Galunisertib (LY2157299) (p<0.01, Figure 1). A similar inhibitory effect on TGF-*β*1 induced proliferation was observed when fibroblasts were preincubated with BAY 41-2272 (p<0.05; Figure 1). Activation of cAMP by forskolin also significantly reduced TGF-*β*1-induced fibroblast proliferation; however, with a lower efficacy compared to BAY 41-2272 (Figure 1). Combining BAY 41-2272 with forskolin completely prevented proliferation (Figure 1). Cell viability was determined by Trypan blue exclusion staining and showed no reduced viability under any treatment (data not shown).

**Figure 2:**
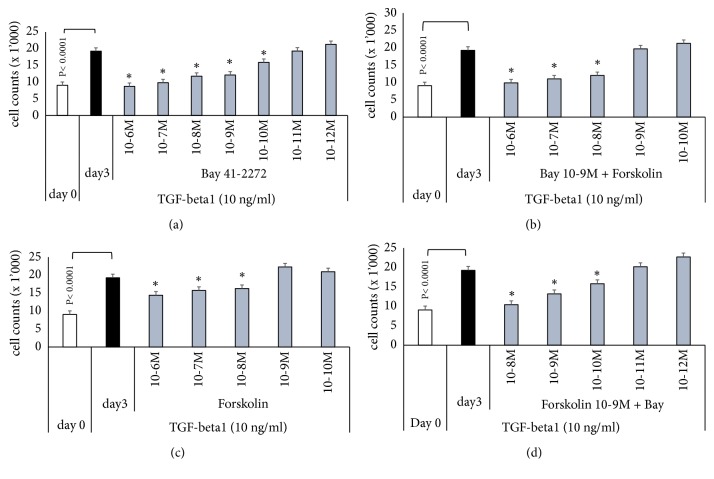
Concentration dependent antiproliferative action of sGC and cAMP activators on TGF-*β*1 induced fibroblast proliferation. (a) Concentration dependent antiproliferative effect of BAY 41-2272 over 3 days; (b) Concentration dependent anti-proliferative effect of forskolin. (c) Supportive effect of forskolin (10^−10^ – 10^−6 ^M) on BAY 41-2272 (10^−9 ^M) inhibition of fibroblast proliferation. (d) Supportive effect of BAY 41-2272 (10^−12^ – 10^−8 ^M) on forskolin (10^−9 ^M) inhibited proliferation. Bars represent mean ±SEM of triplicate experiments. *∗* indicates statistically significant difference (P<0.05) for comparison of TGF-*β*1 stimulation compared to drug effects.

The antiproliferative effect of BAY 41-2272 and forskolin was further investigated in order to determine a possible synergistic effect of the drugs when combined. As shown in Figure 2(a), BAY 41-2272 reduced proliferation in a concentration-dependent manner and the inhibition became significant at concentrations > 10^−10^ M (Figure 2(a)). Forskolin alone reduced proliferation and the effect became significant at concentrations > 10^-8 ^M; however, the effect was not clearly concentration dependent (Figure 2(b)). The maximal inhibitory effect was 47.8%, which was achieved at 10^-6 ^M. When cells were treated with BAY 41-2272 at 10^−9^M combined with forskolin with increasing concentration from 10^−10^ to 10^-6 ^M, the antiproliferative effect was stronger than each drug alone (Figure 2(c)). Vice versa, when cells were treated with forskolin at 10^-9 ^M combined with BAY 41-2272 with increasing concentration from 10^−12^ to 10^-8 ^M, the anti-proliferative effect was increasing in a concentration dependent manner and was stronger compared to each drug alone (Figure 2(d)). None of the antiproliferative drugs described above significantly decreased cell viability as determined by Trypan blue staining (data not shown).

### 3.2. sGC and cAMP Activation Inhibited TGF-*β*1 Induced Synthesis and Deposition of Collagens

**Figure 3:**
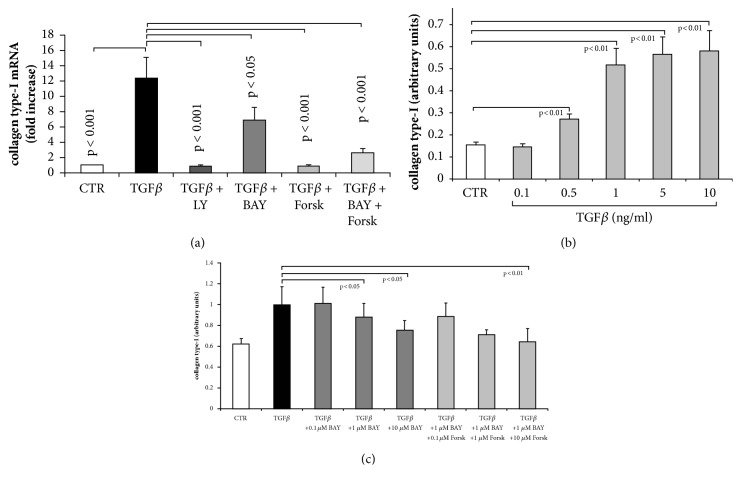
Concentration and time dependent effect of TGF-*β*1 treatment on collagen type-I* de novo *synthesis and deposition in the absence and presence of sGC and cAMP activators. (a) Inhibition of TGF-*β*1 by LY2157299 (10^−6 ^M), or preincubation (30 min) with BAY 41-2272 (10^−6 ^M) or forskolin (10^−6 ^M) significantly reduced transcription of the collagen type-I gene at 24 hours. (b) Concentration dependent induction of collagen type I deposition by TGF-*β*1. (c) Inhibition of collagen type I deposition by increasing concentration of BAY 41-2272 and forskolin in TGF-*β*1 stimulated fibroblasts at 24 hours. Bars represent mean ±SEM of triplicate experiments.

TGF-*β*1 significantly increased the mRNA encoding for collagen type I as shown in Figure 3(a). When preincubated with the TGF-*β*1 inhibitor (LY), the stimulatory effect was completely neutralised. In the presence of BAY 41-2272, the synthesis of collagen type I mRNA was reduced by 52% and in the presence of forskolin by 98%. When BAY 41-2272 was combined with forskolin, the collagen type I mRNA level was reduced by 80%, compared to TGF-*β*1 alone (Figure 3(a)).

On the protein level, TGF-*β*1 dose dependently increased the deposition of collagen type I. This effect became significant at concentration > 0.5 ng/ml (Figure 3(b)). When fibroblasts were preincubated with increasing concentrations with BAY 41-2272, the deposition of collagen type I was significantly reduced at concentration > 1 *μ*M (Figure 3(c)). Adding forskolin to BAY 41-2272 further increased the reducing effect on the TGF-*β*1 stimulated collagen type I deposition (Figure 3(c)). However, the additional effect of forskolin did not become significant compared to BAY 41-2272 alone.

**Figure 4:**
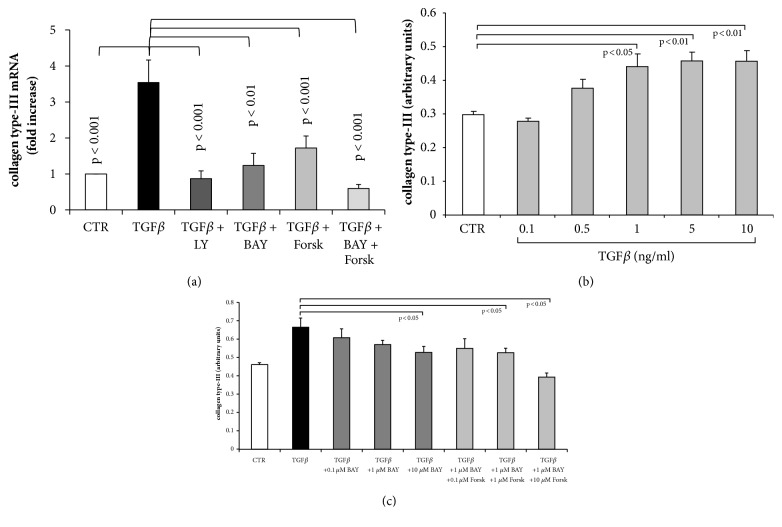
Concentration and time dependent effect of TGF-*β*1 treatment on collagen type III* de novo *synthesis and deposition in the absence and presence of sGC and cAMP activators. (a) Inhibition of TGF-*β*1 by LY2157299 (10^−6 ^M), or preincubation (30 min) with BAY 41-2272 (10^−6 ^M) or forskolin (10^−6 ^M) significantly reduced transcription of the collagen type III gene at 24 hours. (b) Concentration dependent induction of collagen type III deposition by TGF-*β*1. (c) Inhibition of collagen type III deposition by increasing concentration of BAY 41-2272 and forskolin in TGF-*β*1 stimulated fibroblasts at 24 hours. Bars represent mean ±SEM of triplicate experiments.

Depicted in Figure 4(a), the transcription of collagen type III mRNA was significantly increased by TGF-*β*1. Inhibition of TGF-*β*1 (LY) reduced collagen type III mRNA to baseline level and in the presence of BAY 41-27272, a similar effect was achieved (Figure 4(a)). Preincubation with forskolin also reduced the effect of TGF-*β*1 on collagen type III mRNA expression by 58% (Figure 4(a)). Fibroblasts pretreated with the combination of BAY 41-2272 and forskolin did not respond to TGF-*β*1 stimulation, and mRNA expression for collagen type III was at baseline level (Figure 4(a)).

On the protein level, TGF-*β*1 increased collagen type III deposition in a concentration dependent manner achieving significance at concentrations > 1 ng/ml (Figure 4(b)). Preincubation with BAY 41-2272 reduced TGF-*β*1 induced collagen type III deposition in a concentration dependent manner, which achieved significance only at 10 *μ*M (Figure 4(c)). The combination of BAY 41-2272 and forskolin was more effective as shown in Figure 4(c).

**Figure 5:**
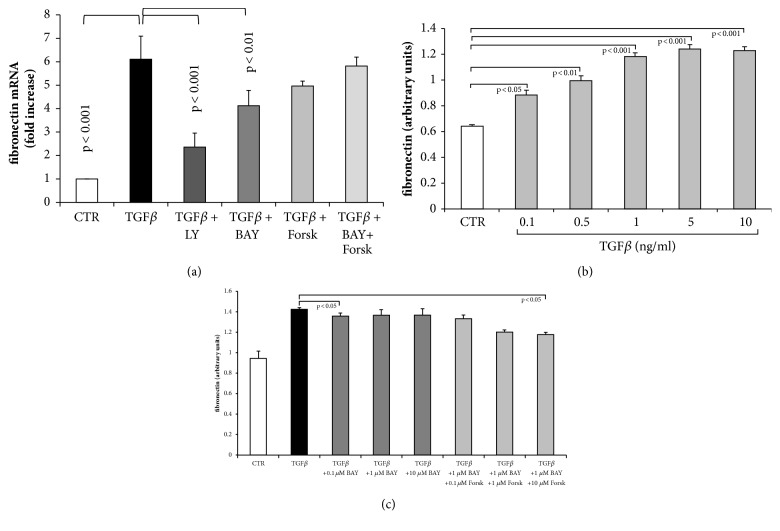
Concentration and time dependent effect of TGF-*β*1 treatment on fibronectin* de novo *synthesis and deposition in the absence and presence of sGC and cAMP activators. (a) Inhibition of TGF-*β*1 by LY2157299 (10^−6 ^M), or preincubation (30 min) with BAY 41-2272 (10^−6 ^M) or forskolin (10^−6 ^M) significantly reduced transcription of the fibronectin gene at 24 hours. (b) Concentration dependent induction of fibronectin deposition by TGF-*β*1. (c) Inhibition of fibronectin deposition by increasing concentration of BAY 41-2272 and forskolin in TGF-*β*1 stimulated fibroblasts at 24 hours. Bars represent mean ±SEM of triplicate experiments.

TGF-*β*1 significantly upregulated the expression of fibronectin mRNA in human lung fibroblasts (Figure 5(a)). Inhibiting TGF-*β*1 by LY2157299 reduced fibronectin mRNA by 82%, but did not completely block the effect of TGF-*β*1 (Figure 5(a)). BAY 41-2272 reduced fibronectin mRNA in fibroblasts stimulated with TGF-*β*1 by only 33% (Figure 5(a)). Forskolin had no significant reducing effect on TGF-*β*1 induced fibronectin mRNA expression and when combined with BAY 41-2272, this effect was even lower (Figure 5(a)).

TGF-*β*1 increased fibronectin deposition in a concentration dependent manner and the effect was significant at concentrations > 0.1 ng/ml (Figure 5(b)). In the presence of BAY 41-2272, the TGF-*β*1 induced deposition of fibronectin was only marginally reduced (Figure 5(c)). However, when combined with forskolin, the deposition of fibronectin was reduced and achieved significance at the highest concentrations of both drugs (Figure 5(c)).

### 3.3. BAY 41-2272 Reversed TGF-*β*1 Induced *α*-SMA and *β*-Catenin Expression and Structure

**Figure 6:**
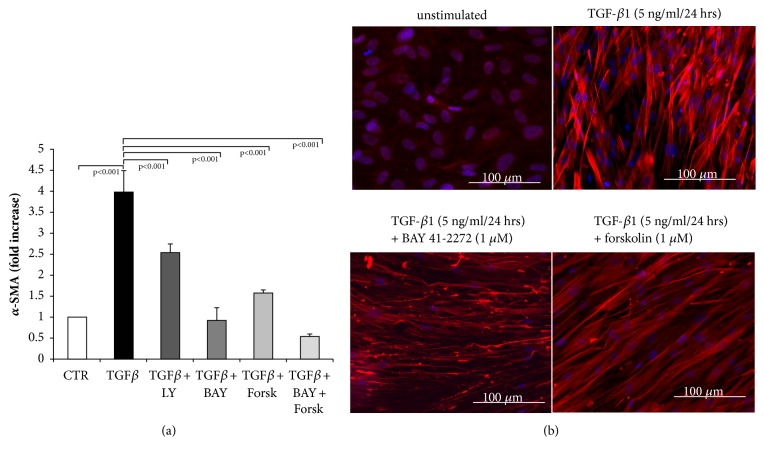
Blockade of myofibroblasts differentiation by sGC and cAMP activation. (a) Transcription of *α*-SMA was inhibited by LY2157299, BAY 41-2272 and forskolin (all at 10^−6 ^M) in TGF-*β*1 stimulated fibroblasts. Bars represent mean ±SEM of triplicate experiments. (b) Representative immunochemical staining for *α*-SMA in unstimulated cells (left upper panel), TGF-*β*1 (5 ng/ml) stimulated fibroblasts (right upper panel), and cells preincubated (30 min) with increasing concentrations of either BAY 41-2272 or forskolin followed by TGF-*β*1-stimulation (5 ng/ml). Similar results were obtained in three additional experiments.

TGF-*β*1 induced the expression of mRNA encoding for *α*-SMA, and this effect was reduced by 35% in the presence of the TGF-*β*1 inhibitor (LY) as shown in Figure 6(a). BAY 41-2272 reduced the mRNA level of *α*-SMA in TGF-*β*1 stimulated fibroblasts to baseline level (Figure 6(a)). Forskolin alone reduced *α*-SMA mRNA expression by 80%, and when combined with BAY 41-2272, it completely prevented TGF-*β*1 induced *α*-SMA mRNA expression (Figure 6(a)).

Unstimulated fibroblasts expressed a low level of *α*-SMA in the cytosol as shown in Figure 6(b). TGF-*β*1 significantly increased the expression of *α*-SMA and changed the confirmation of the protein into contractile fibrils (Figure 6(b)). When preincubated for 30 minutes with BAY 41-2272, the effect of TGF-*β*1 on the expression and arrangement of *α*-SMA was prevented (Figure 6(b)). Forskolin had a less prominent reducing effect on both expression and arrangement of *α*-SMA (Figure 6(b)).

**Figure 7:**
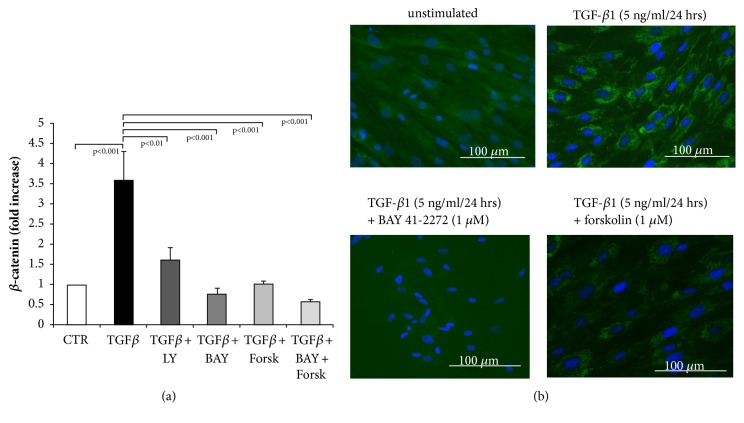
Blockade of *β*-catenin expression in fibroblasts by sGC and cAMP activation. (a) Transcription of *β*-catenin was inhibited by LY2157299, BAY 41-2272, and forskolin (all at 10^−6 ^M) in TGF-*β*1 stimulated fibroblasts. Bars represent mean ±SEM of triplicate experiments. (b) Representative immunochemical staining for *β*-catenin in unstimulated cells (left upper panel), TGF-*β*1 (5 ng/ml) stimulated fibroblasts (right upper panel), and cells preincubated (30 min) with increasing concentrations of either BAY 41-2272 or forskolin followed by TGF-*β*1-stimulation (5 ng/ml). Similar results were obtained in three additional experiments.

The expression of *β*-catenin mRNA was significantly increased by TGF-*β*1 within 24 hours, and this effect was nearly completely prevented in the presence of TGF-*β*1 inhibitor (Figure 7(a)). Similarly the preincubation of fibroblasts with BAY 41-2272, or forskolin, or the combination of both was effective; the mRNA level of *β*-catenin in TGF-*β*1 stimulated fibroblasts was reduced to baseline level by each of the treatments (Figure 7(a)).

As shown by immunochemistry, *β*-catenin was expressed at a higher level in the cytosol of fibroblasts stimulated with TGF-*β*1, compared to unstimulated fibroblasts (Figure 7(b)). The expression of *β*-catenin was significantly reduced in fibroblasts that were preincubated either BAY 41-2272 or forskolin.

## 4. Discussion

IPF is characterised by the accumulation of fibroblasts and increased ECM deposition, which resembles features of scar formation, and were assigned as fibrotic foci [25]. Both events are assumed to result from increased levels of TGF-*β*1, which were described in fibrotic tissues of IPF patients [26]. The experiments described above confirmed the antifibrotic effect of cAMP activation by forskolin, as well as a novel antiproliferative effect or BAY 41-2272, which is dependent on the activation of sGC. The combination of both classes of drugs showed an improved effect to each drug alone; however, the effect was additive rather than synergistic. In regard to ECM deposition, the presented data indicates that neither of the drugs has a general inhibitory effect and it is necessary to assess the drug effect on each component of the ECM. Furthermore, the presented data suggest that sGC combined with cAMP activation reduces the development of myofibroblasts. The above data indicate that sGC such as BAY 41-2272 may help to reduce the progression of fibrosis.

In IPF, increased proliferation of local resident lung fibroblasts has been suggested to result in fibrotic foci formation [27]. In addition, epithelial to mesenchymal transition (EMT) was reported to cause the same pathologies [9, 28]. Both proliferation and EMT have been linked to the increased activity of TGF-*β*1 in IPF [29, 30] and involved Wnt and *β*-catenin signalling [31]. In human embryonic pulmonary fibroblasts, it was reported that Wnt and *β*-catenin activation led to pathological features resembling IPF [32]. In an animal model, Wnt signalling was essential for TGF-*β*1 induced myofibroblasts generation [33]. Inhibition of *β*-catenin reduced EMT in an immortalised cell line, A549, which was used as a model for pulmonary fibrosis [34]. Furthermore, inhibition of Wnt signalling supported the engraftment of mesenchymal stem cells into damaged lung tissues and therefore improved tissue repair [35]. The observation that sGC and cAMP activity downregulated TGF-*β*1 induced *β*-catenin and *α*-SMA indicates that the combined drugs have the potential to limit myofibroblasts generation. Moreover, BAY 41-2272 prevented the arrangement of *α*-SMA in contractile fibrils, suggesting a reduced formation of contractile myofibroblasts. However, the combination of both drugs did not enhance this effect of BAY 41-2272. The above presented data showed that BAY 41-2272 and forskolin, are potent inhibitors of *β*-catenin and therefore may present novel therapeutic options for IPF. However, Wnt and *β*-catenin signalling are essential for the development and the function of the lung and its role in the pathogenesis of IPF has to be further studied [36].

The role of G-protein coupled receptors in the pathogenesis of fibrotic disease had been suggested in animal models earlier [37]. In a recent study, G-protein coupled receptors activity reduced fibroblast proliferation and differentiation by increasing cAMP [38]. However, the authors concluded that the level of cAMP generated by G-protein coupled receptors is not indicator of drug's efficacy and seems to follow a “yes” or “no” pattern. This observation was in part confirmed by the results displayed above, showing a sudden increase of inhibitory effects at concentrations > 10^−8^ M for forskolin.

With regard to IPF therapy, nintedanib and pirfenidone achieved their beneficial effects in part by inhibiting TGF-*β*1 [39, 40]. However, both drugs have severe side effects and there is a need of alternative therapy options. One of such drug candidates is BAY 41-2272, which has been reported to prevent fibrosis in rat hearts [41]. The same drug had antiproliferative effects in vascular smooth muscle cells [42, 43]. BAY 41-2272 achieved its antiproliferative effects through the activation of cAMP and cGMP dependent protein kinases [43]. The data presented above show that BAY 41-2272 reduces fibroblasts proliferation in a concentration dependent manner, suggesting it may be a drug candidate for IPF treatment.

Fibrotic foci in IPF tissue are characterised by increased deposition of ECM, which is composed differently compared to ECM in the healthy lung [9, 13, 26]. The change in the composition of the ECM results from an increased deposition of collagens types I and III in the fibrotic foci and an overall increase of fibronectin. Nintedanib and Pirfenidone have the potential to reduce the accumulation of collagens and fibronectin in isolated cells; however, such an effect has not been proven in patient samples [7, 8, 39, 44]. The above presented data shows that sGC, such as BAY 41-2272, may have a similar effect on reducing TGF-*β*1 induced* de novo* deposition of collagen types I and III. However, neither BAY 41-2272 nor forskolin had a significant effect on TGF-*β*1 induced fibronectin deposition, except at the highest concentration used in this study. Fibronectin has recently been indicated to play an essential role in wound repair and specifically in the recovery and function of epithelial cells [45]. Therefore, the lack of action of BAY 41-2272 on TGF-*β*1 induced fibronectin deposition may be regarded as beneficial and restore the function of the epithelial cells in IPF.

The presented data indicates that sGC presents a novel class of drug for IPF therapy; however, this study was limited by the fact that the drugs were only investigated in nondiseased human lung fibroblasts and therefore no information was available if they achieve the same effect as diseased fibroblasts. We did not consider including animal models, since bleomycin-induced fibrosis does not represent the pathogenesis leading to IPF. Future studies are needed to clarify the beneficial effect of sGC on wound repair and tissue regeneration in IPF.

## 5. Conclusions

The data presented in this study indicates that BAY 41-2272, a sGC activator, may be considered as a novel therapeutic option for IPF. Furthermore, it may be beneficial to combine sGC activator with forskolin to improve their antifibrotic potential.

## Data Availability

The data used to support the findings of this study are available from the corresponding author upon request.
